# A Cell-Adapted Live-Attenuated Vaccine Candidate Protects Pigs against the Homologous Strain VNUA-ASFV-05L1, a Representative Strain of the Contemporary Pandemic African Swine Fever Virus

**DOI:** 10.3390/v15102089

**Published:** 2023-10-13

**Authors:** Quang Lam Truong, Lihua Wang, Tuan Anh Nguyen, Hoa Thi Nguyen, Son Danh Tran, Anh Thi Vu, Anh Dao Le, Van Giap Nguyen, Phuong Thi Hoang, Yen Thi Nguyen, Thi Luyen Le, Thang Nguyen Van, Thi My Le Huynh, Huong Thi Lan Lai, Rachel Madera, Yuzhen Li, Jishu Shi, Lan Thi Nguyen

**Affiliations:** 1 Key Laboratory of Veterinary Biotechnology, Faculty of Veterinary Medicine, Vietnam National University of Agriculture, Gia Lam, Ha Noi 12406, Vietnam; anhanhtuan997@gmail.com (T.A.N.); hoanguyen2405@gmail.com (H.T.N.); trandanhson001@gmail.com (S.D.T.); vuanhhd98@gmail.com (A.T.V.); daoleanhvetlab@gmail.com (A.D.L.); htphuong1989@gmail.com (P.T.H.); yennt212@gmail.com (Y.T.N.); leluyentyc@gmail.com (T.L.L.); nguyenvanthang22394@gmail.com (T.N.V.); ltlhuong@vnua.edu.vn (H.T.L.L.); 2 Center on Vaccine Evaluation and Alternatives for Antimicrobials, Department of Anatomy and Physiology, College of Veterinary Medicine, Kansas State University, Manhattan, KS 66506, USA; lihua@vet.k-state.edu (L.W.); rachelmadera@vet.k-state.edu (R.M.); yuzhen@vet.k-state.edu (Y.L.); 3 Department of Veterinary Microbiology and Infectious Diseases, Faculty of Veterinary Medicine, Vietnam National University of Agriculture, Gia Lam, Ha Noi 12406, Vietnam; nvgiap@vnua.edu.vn (V.G.N.); huynhtmle@vnua.edu.vn (T.M.L.H.)

**Keywords:** African swine fever, African swine fever virus, cell adapted, live attenuated, vaccine, immunity, protection

## Abstract

African swine fever (ASF) is a lethal and highly contagious transboundary animal disease with the potential for rapid international spread. Currently, there is no ASF vaccine commercially available. All infected animals must be isolated and culled immediately upon the confirmation of the presence of the virus. Studies leading to the rational development of protective ASF vaccines are urgently needed. Here, we generated a safe and efficacious live-attenuated vaccine (LAV) VNUA-ASFV-LAVL2 by serially passaging a field isolate (VNUA-ASFV-05L1, genotype II) in porcine alveolar macrophages (PAMs, 65 passages) and an immortalized porcine alveolar macrophage cell line (3D4/21, 55 passages). VNUA-ASFV-LAVL2 can efficiently replicate in both PAMs and 3D4/21 cells. It provides 100% protection, even with the low dose of 10^2^ HAD_50_, to the vaccinated pigs against the challenge of contemporary pandemic ASFV field isolate. Pigs vaccinated with this LAV in a dose range of 10^2^ to 10^5^ HAD_50_ remained clinically healthy during both the 28-day observation period of immunization and the 28-day observation period of challenge. VNUA-ASFV-LAVL2 was eliminated from blood by 28 days post-inoculation (DPI), and from feces or oral fluids by 17 DPI. Although the vaccine strain in serum remained a safe and attenuated phenotype after five passages in swine, a reversion-to-virulence study using blood or tissue homogenates at peak viremia will be conducted in the future. ASFV-specific IgG antibodies and significant cellular immunity were detected in vaccinated pigs before the ASFV challenge. These results indicate that the VNUA-ASFV-LAVL2 strain is a safe and efficacious LAV against the genotype II ASFV strain responsible for current ASF outbreaks in Asia.

## 1. Introduction

African swine fever (ASF) is a highly contagious and severe hemorrhagic transboundary swine viral disease with up to 100% mortality rate, leading to a tremendous socio-economic loss worldwide [1]. The causative agent, ASF virus (ASFV), classified under the *Asfarviridae* family and *Asfivirus* genus, is a large, enveloped virus containing a double-stranded DNA (dsDNA) genome of approximately 170–190 kilobase pairs (kbp) [2]. A total of 24 ASFV genotypes (I-XXIV) have been described based on the ASFV p72 major capsid protein gene (*B646L*) [3]. The highly virulent ASFV genotype II that emerged in the Caucasus region in 2007 is responsible for the contemporary pandemic in Europe/Asia, and the outbreaks in Caribbean countries (Dominican Republic and Haiti) [4,5,6,7]. The first outbreak of ASF in Vietnam was reported in early 2019 and quickly spread across the entire country with more than 8 million piglets depopulated, which was equal to nearly 25 percent of the total pig population in 2020 [8,9,10]. At present, the ASFV genotype II has become endemic, and the ASF outbreak is continuing to occur frequently in Vietnam, raising the greatest concerns not only for the government but also for the pig industries.

The clinical presentation and the gross pathological lesions of ASF in domestic pigs may vary depending on the virulence of the virus isolate, the route, the dose of infection, and host characteristics. For acute ASF, the clinical course is characterized by high fever, with temperatures up to 42 °C, lethargy, anorexia, and inactivity [11]. The lack of safe and efficacious ASF vaccines is the greatest challenge in the prevention and control of ASF. In the past several years, extensive efforts have been taken to develop ASF vaccines that include inactivated vaccines, recombinant subunit vaccines (protein-based, DNA, viral-vectored), and live-attenuated strains (LAVs) [11,12,13,14]. Up to now, the inactivated and recombinant subunit vaccines have not yet been shown to be efficacious [12,15,16,17]. In contrast, recent promising results with LAVs provide hope for a safe and efficacious vaccine against ASF. Several groups have developed LAVs by the deletion of genes associated with virulence, which induce solid protective immunity against homologous strains. Among them, ASFV-G-ΔI177L, ASFV-G-ΔI177LΔLVR, and ASFV-G-ΔMGF strains proved to be an attenuated phenotype in pigs and conferred full protection against the challenge of the parental ASFV-Georgia strain [18,19,20,21,22]. However, ideal LAVs that meet the commercial vaccine demands still face challenges related to stable cell lines for producing the LAV at a large scale, and their efficacy depends on the age of pigs, reversion-to-virulence, vaccine virus shedding, and differentiation of infected from vaccinated animals (DIVA) [13,14,23,24].

LAVs, created by passaging a virus in cultured cells, have proven to be an effective means for preventing many viral diseases, including smallpox, polio, measles, mumps, and yellow fever. Those attenuated vaccines elicit strong immunoprotective cellular and antibody responses, and often confer lifelong immunity with only one or two doses [25]. However, there is currently no report of using this technology to develop ASF LAVs. Since the identification of ASF outbreak in Vietnam, research towards vaccine development using field ASFV genotype II isolate has been initiated in our groups. Here, we report the generation of a safe and efficacious LAV vaccine VNUA-ASFV-LAVL2 from a field isolate using cell passage. VNUA-ASFV-LAVL2 can not only provide 100% protection to pigs against virulent contemporary pandemic ASFV infection, but also it can efficiently replicate in the commercially available 3D4/21 cell line. 

## 2. Materials and Methods

### 2.1. Animals

Piglets (4–7 weeks old) that tested negative for ASFV and ASFV-antibody were obtained from clean pig farms and used for experiments in this study. The pigs were fed a standard commercial diet. In Vietnam, the pigs were housed in the Animal Biosafety Research Facility of the Faculty of Veterinary Medicine, Vietnam National University of Agriculture (VNUA). In the United States, the pigs were housed under laboratory biosafety level III agriculture (BSL3-Ag) conditions at the Biosecurity Research Institute (BRI), Kansas State University (KSU). Animal care and protocols were approved by Institutional Animal Care and Use Committee at Vietnam National University of Agriculture (VNUA-2021/01) and at Kansas State University (IACUC#4845). All animal experiments were conducted strictly adhering to the IACUC protocols. 

### 2.2. Cells and Virus

Primary pulmonary alveolar macrophages (PAMs) were prepared as described previously [26]. PAMs were maintained in a medium containing Dulbecco’s modified Eagle medium (DMEM, Life Technologies, Grand Island, NY, USA) supplemented with 10% heat-inactivated fetal bovine serum (FBS, Thermo Scientific, Waltham, MA, USA) and Antimycotic (Life Technologies, Grand Island, NY, USA) at 37 °C in 5% CO_2_ incubator. 

3D4/21 (immortalized porcine alveolar macrophage cell line, ATCC, CRL-2843) cells were cultured in high-glutamine RPMI medium (RPMI, Life Technologies, Grand Island, NY, USA) adjusted to contain 1.5 g/L sodium bicarbonate (Thermo Scientific, Waltham, MA, USA), 4.5 g/L glucose (Thermo Scientific, Waltham, MA, USA), 10 mM HEPES (Thermo Scientific, Waltham, MA, USA), 1.0 mM sodium pyruvate (Thermo Scientific, Waltham, MA, USA) supplemented with 1% MEM Non-Essential Amino Acids Solution (Thermo Scientific, Waltham, MA, USA), and 10% heat-inactivated fetal bovine serum (FBS, Thermo Scientific, Waltham, MA, USA) at 37 °C in 5% CO_2_ incubator. 

Virulent VNUA-ASFV-05L1 strain (genotype II) was isolated from the spleen of a domestic pig with typical acute ASF during an ASF outbreak in Northern Vietnam in 2020 [27]. It is maintained in BSL-3 laboratories of Vietnam National University and Kansas State University. This virus was used for the generation of LAV and the challenge studies conducted on pigs in this study.

### 2.3. In Vitro Evaluation of Virus Passage and Virus Replication

Wild-type virulent VNUA-ASFV-05L1 strain was passaged in PAM cells and 3D4/21 cells. We used the same culture medium for infection and passage of VNUA-ASFV-05L1 strain in PAM cells. For 3D4/21 cells, we added 1.25% dimethyl sulfoxide (DMSO) in the culture medium for improving its ability to support VNUA-ASFV-05L1 replication. Monolayers were infected with VNUA-ASFV-05L1 at the multiplicity of infection (MOI) of 1 and incubated for 4 days. Culture supernatant was then harvested, titrated, and passaged onto fresh monolayers at MOI of 1. The VNUA-ASFV-05L1 was passaged 65 times in PAM cells first and then 55 times in 3D4/21 cells to generate VNUA-ASFV-LAVL2. Virus titers in the supernatants at each passage were titrated in PAMs. PAMs were pre-seeded (80–100% confluent) and incubated with 10-fold dilutions of the harvested supernatants. After 2 h incubation, 2% porcine red blood cells were added for hemadsorption (HAD) testing. After four days culture, the presence of ASFV was assessed using HAD under an inverted microscope. HAD_50_ was calculated using the method of Reed and Muench [28].

For testing the in vitro replication characteristics of VNUA-ASFV-LAVL2 and its parental virus (VNUA-ASFV-05L1), monolayers of PAMs and 3D4/21 cells at 90% confluency in 24-well culture plates were infected with the viruses at MOI of 1. After 2 h incubation, the inoculum was removed. Cells were washed and replaced with fresh culture media. Cultures (including cells and culture medium) were collected at 0, 24, 36, 48, 60, 72, 84, and 96 h post-infection (HPI). The collected cultures were subjected to three freeze–thaw cycles. After centrifuging the cell debris, virus titers in the supernatant were tested and calculated as described above. All experiments were performed in duplicate. 

### 2.4. Safety Testing of LAV in Pigs

To test the safety of VNUA-ASFV-LAVL2 in pigs, five groups of pigs (n = 5/group) were inoculated intramuscularly (i.m.) either with 10^2^, 10^3^, 10^4^, and 10^5^ HAD_50_/dose of VNUA-ASFV-LAVL2 or with 8 × 10^2^ HAD_50_ of parental VNUA-ASFV-05L1. Control pigs (n = 3) were inoculated (i.m.) with DMEM medium. Blood, oral fluids, rectal swabs, and serum samples of pigs were collected at 0, 3, 5, 7, 9, 11, 14, 17, 21, 24, and 28 days post-inoculation (DPI). The presence of clinical signs (anorexia, depression, fever, purple skin discoloration, staggering gait, diarrhea, and cough), body temperature, and survival rate were recorded daily throughout the experiment by qualified personnel [18]. 

To further evaluate the safety of the LAV, we pooled the serum of pigs inoculated with 10^3^ HAD_50_/dose of VNUA-ASFV-LAVL2, and we i.m. inoculated 1 mL/dose in pigs (n = 3) to obtain passage 1 (P1). At 9 DPI, when the serum samples showed the highest Ct value with ASFV real-time PCR (RT-PCR), we pooled the collected serum samples from P1 pigs and i.m. inoculated 1 mL/dose in pigs (n = 3) to obtain P2. Five passages were performed with the same method and procedure. Blood and serum samples were collected at 3, 5, 7, 9, 11, 14, 17, 21, 24, 28, and 35 DPI. Clinical signs (anorexia, depression, fever, purple skin discoloration, staggering gait, diarrhea, and cough), body temperature, and survival rate were recorded daily throughout the experiment. 

### 2.5. Efficacy Evaluation of LAV in Pigs

At 28 DPI, pigs in groups inoculated with 10^2^, 10^3^, 10^4^, and 10^5^ HAD_50_/dose of VNUA-ASFV-LAVL2 and in DMEM-inoculated control group were challenged (i.m.) with 1 × 10^3^ HAD_50_ of parental VNUA-ASFV-05L1. The blood, oral fluids, rectal swabs, and serum samples of pigs were collected at 0, 3, 5, 7, 9, 11, 14, 17, 21, 25, and 28 days post-challenge (DPC). The presence of clinical signs, body temperature, and survival rate were recorded daily throughout the experiment. The dead pigs in the control group were assessed for typical signs of ASFV pathological lesions. Tissue samples (1.5 g) from inner part of brain, kidney, liver, spleen, lung, heart, lymph nodes, stomach, small intestine, large intestine, and bone marrow were aseptically collected from all dead pigs during the experiment, and pigs were euthanized at 28 DPC by qualified personnel. 

In a separate animal study, to evaluate the efficacy of LAV against challenge of high doses of ASFV, 10 pigs (n = 5/group) were inoculated (i.m.) with 10^2^ or 10^3^ HAD_50_/dose of VNUA-ASFV-LAVL2. Control pigs (n = 3) were i.m. inoculated with DMEM. In addition, we housed 2 contact pigs of each vaccinated pig group in the same cage. At DPI 28, a 10 times higher dose (8 × 10^3^ HAD_50_/dose) than the standard challenge dose (8 × 10^2^ HAD_50_/dose) of parental VNUA-ASFV-05L1 was used to challenge the immunized and control pigs. Blood, oral fluids, rectal swabs, and serum samples of pigs were collected at 0, 3, 5, 7, 9, 11, 14, 17, 21, 25, and 28 days post-inoculation (DPI). The presence of clinical signs (anorexia, depression, fever, purple skin discoloration, staggering gait, diarrhea, and cough), body temperature, and survival rate were recorded daily throughout the experiment. 

### 2.6. DNA Extraction, Quantitative ASFV Real-Time PCR, and Genome Sequencing

Quantitative RT-PCR was conducted to detect ASFV DNA in oral fluid and rectal swabs, blood, and tissue homogenates of the experimental pigs. DNA was extracted using an automated King Fisher^TM^ Duo Prime DNA/RNA extraction system (Thermo Fisher Scientific, Waltham, MA, USA) with MagMAX CORE nucleic acid purification kit (Life Sciences, New York, NY, USA), according to the manufacturer’s protocols. ASFV DNA was then detected using Platinum SuperMix-UDG kit (Invitrogen, Waltham, MA, USA) on CFX Optus 96 Real-time PCR system (BioRad, Hercules, CA, USA) using p72 primers and the probe developed by Haines et al. [29]. Samples with Ct values <40 were considered to be positive. 

The ASFV genome next-generation sequencing and de novo genome assembly were performed as described previously [27]. LAV whole genome analysis and comparison with the parental virus VNUA-ASFV-05L1 (GenBank accession number MW465755.1) were performed using Genome Workbench from NCBI (https://www.ncbi.nlm.nih.gov/tools/gbench/, accessed on 1 September 2023.) and CLC Sequence Viewer 8.0.0 (Qiagen, Hilden, Germany).

### 2.7. ASFV-Specific Antibody Detection

The ASF blocking ELISA kit (INGEZIM PPA COMPAC 11.PPA.k3, Ingenasa, Madrid, Spain) was used to detect specific anti-ASFV antibodies in serum samples. The procedure of the commercial ELISA kit was performed as described in the manufacturer’s instructions. For each sample, the competition percentage (S/N%) was calculated according to the manufacturer’s instructions, and more than or equal to (≥) 50% was considered positive, between 40 and 50% was considered doubtful, and ≤40% was considered negative.

### 2.8. ELISPOT and ELISA to Evaluate IFN-γ and IL-10 Cellular Responses in Pigs

Interferon gamma (IFN-γ) production by peripheral blood mononuclear cells (PBMC) of pigs was determined using enzyme-linked immunospot assay (ELISPOT). Briefly, the PBMCs of pigs were freshly isolated using density-gradient centrifugation with 1.077 g/mL Ficoll-Paque PREMIUM density gradient media (Cytiva, Marlborough, MA, USA) in SepMate^TM^ tubes (STEMCELL Technologies Inc., Cambridge, MA, USA). The PBMCs’ suspensions (2 × 10^5^/well) were added to MultiScreenHTS IP Filter Plates (Millipore Sigma, Lenexa, KS, USA) precoated with Mouse anti-pig IFNγ (BD Biosciences, San Jose, CA, USA). The PBMCs were incubated 18 h at 37 °C with 100 μL/well of stimulatory agents: phorbol myristate acetate (PMA, 25 ng/mL)/ionomycin (2.5 μg/mL) (Millipore Sigma, Lenexa, KS, USA) combination as positive control; 100 μL/well of VNUA-ASFV-05L1 (10^5^ HAD_50_/mL) as re-stimulation agent; and 100 μL/well of complete culture media as unstimulated control. After washing, Biotin mouse anti-pig IFNγ (BD Biosciences, San Jose, CA, USA), HRP Streptavidin for ELIspot (BD Biosciences, San Jose, CA, USA), and fresh NovaRED Peroxidase Substrate (Vector Labs, Newark, CA, USA) were added according to the manufacturer’s instructions. Finally, the reaction was stopped by rinsing the plate with deionized water. The number of spots was determined using a CTL Spot Reader (CTL, New York, NY, USA). 

The concentration of porcine IL-10 in serum samples of pigs was measured using porcine IL-10 Quantikine ELISA kit (Thermo Fisher Scientific, Waltham, MA, USA) according to the manufacturer’s instructions. 

### 2.9. Statistical Analysis

Statistical analysis was performed using GraphPad Prism 6.0 (GraphPad Software, San Diego, CA, USA). The data from assays for virus titration in cell cultures and blood samples in experimental piglets at different time points and efficacy studies were expressed as the mean log HAD ± SD (Standard deviation) for each group and analyzed with Student’s *t*-test. The data for antibody and cellular responses were expressed as mean readings ± SD for each group. The significance of differences between the experimental groups was analyzed with analysis of variance (ANOVA) followed by Turkey’s post-test. For all statistical analyses, *p* values less than 0.05 were considered as statistically significant.

## 3. Results

### 3.1. VNUA-ASFV-LAVL2 Replicates Stably and Efficiently in Both PAMs and 3D4/21 Cells

**Figure 1:**
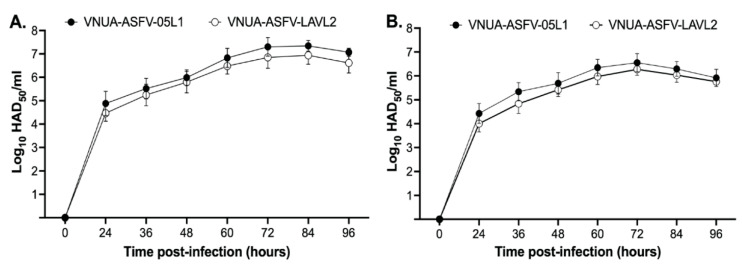
Growth curves of the VNUA ASFV-LAVL2 (passage 120) and parental VNUA-ASFV-05L1 in (**A**) PAMs and (**B**) 3D4/21 cells.

We compared the growth characteristics of VNUA-ASFV-LAVL2 with its parental virus VNUA-ASFV-05L1 in PAMs and 3D4/21 cells. VNUA-ASFV-LAVL2 showed growth at moderate titers of around 10^5^ HAD_50_/mL for the first 15 passages, and then at moderate-to-high titers of around 10^5^ to 10^6^ HAD_50_/mL in 3D4/21 cells. The virus retained this stable replication phenomenon (with similar growth ability) from passage 110 to passage 120 with the highest titer 10^6^ HAD_50_/mL at 72 HPI. Passage 120 showed comparable growth kinetics with less than 0.5 log_10_ at each time points post-infection in both PAMs and 3D4/21 cells compared to that of parental VNUA-ASFV-05L1 strain (Figure 1A,B).

### 3.2. Genome Comparison of VNUA-ASFV-LAVL2 with Parental Virus VNUA-ASFV-05L1

Next-generation sequence analysis data of VNUA-ASFV-LAVL2 (passage 120) showed that the whole genome of VNUA-ASFV-LAVL2 contains 175,085 nucleotides and has 38.8% GC content. It lacked a region corresponding to the multigene family (MGF) between 168,120 bp and 179,264 bp of VNUA-ASFV-05L1 (GenBank accession number MW465755.1), resulting in 13 gene deletions (MGF110-5-6L, MGF110-7L, 285L, MGF 110-8L, MGF 100-1R, MGF 110-9L, MGF 110-11L, MGF 110-14L, MGF 110-12L, MGF 110-13L, MGF360-4L, and MGF360-6L, X69R) and 14 uncharacterized sequence deletions (Table 1). 

Mutations that resulted in amino acid substitutions or protein disruptions are found in the genome of VNUA-ASFV-LAVL2 as well (Table 1). 

### 3.3. VNUA ASFV-LAVL2 Is Significantly Attenuated and Highly Safe in Pigs

**Figure 2:**
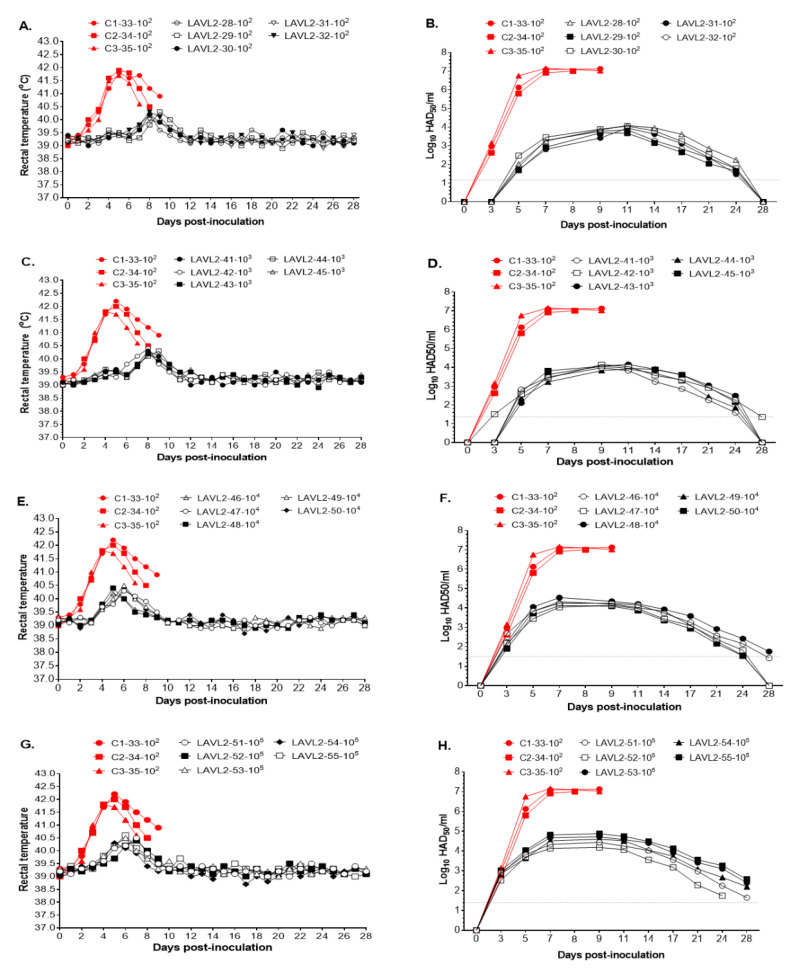
Rectal temperature and viremia of VNUA-ASFV-LAVL2-vaccinated pigs and parental VNUA-ASFV-05L1-inoculated control pigs. Daily rectal temperature of control pigs and pigs vaccinated with the following doses of VNUA-ASFV-LAVL2: (**A**) 10^2^, (**C**) 10^3^, (**E**) 10^4^, and (**G**) 10^5^ HAD_50_. Daily viremia of control pigs and pigs vaccinated with the following doses of VNUA-ASFV-LAVL2 strain: (**B**) 10^2^, (**D**) 10^3^, (**F**) 10^4^, and (**H**) 10^5^ HAD_50_. The dotted line represents the limit of detection.

Pigs vaccinated with VNUA-ASFV-LAVL2 from low dose (10^2^ HAD_50_) to high dose (10^5^ HAD_50_) showed transient low fever (<40.6 °C) by 7–10 DPI (peak at 8–9 DPI) (Figure 2A,C) and 5–7 DPI (peak at 5–7 DPI) (Figure 2E,G), and then showed normal body temperature during the observation period of 28 days. No obvious ASFV-specific clinical signs were observed for the vaccinated pigs. In contrast, the parental VNUA-ASFV-05L1-inoculated control pigs displayed clinical signs of ASF, including high fever, anorexia, cough, depression, staggering gait, and diarrhea. All control pigs died due to severe clinical symptoms during 8–10 DPI. 

VNUA-ASFV-LAVL2-vaccinated pigs showed significantly lower viremia than parental VNUA-ASFV-L1-inoculated control pigs. The control pigs reached peak titers of approximately 10^7^ HAD_50_/mL before they died, whereas VNUA-ASFV-LAVL2-vaccinated pigs reached the highest viremia titer of 10^4^ to 10^5^ HAD_50_/mL at 7 to 11 DPI (peak at 11 DPI for low dose, and at 7 DPI for high dose), and then these titers rapidly declined and almost cleared at 28 DPI (Figure 2B,D,F,H).

**Figure 3:**
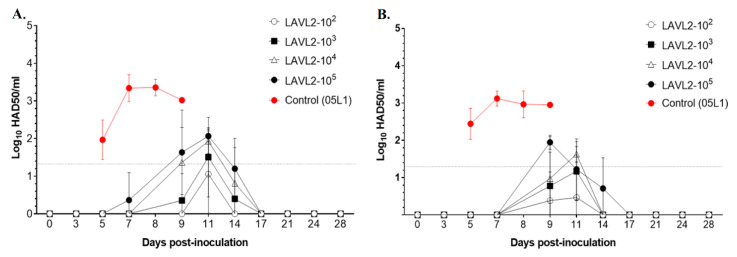
Viral titers in (**A**) oral fluids swabs and (**B**) rectal swabs of VNUA-ASFV-LAVL2-vaccinated pigs and parental VNUA-ASFV-05L1-inoculated control pigs. Data are presented as mean ± SD for pigs in each group. The dotted line represents the limit of detection.

In oral fluid swabs (Figure 3A) and rectal swabs (Figure 3B), only very low viral titers (less than 10^2^ HAD_50_/mL) were detected in VNUA-ASFV-LAVL2-vaccinated pigs from 9 DPI to 14 DPI, and the VNUA-ASFV-LAVL2 viruses were rapidly cleared after 17 DPI. In contrast, for the VNUA-ASFV-05L1-inoculated control pigs, viral titer in oral fluid swabs and rectal swabs reached peak titers of approximately 3.5 × 10^3^ HAD_50_/mL and remained steady before they died. 

### 3.4. VNUA-ASFV-LAVL2 Maintained Safe and Attenuated Phenotype When Passaged in Pigs Determined with Pooled Serum Samples

**Figure 4:**
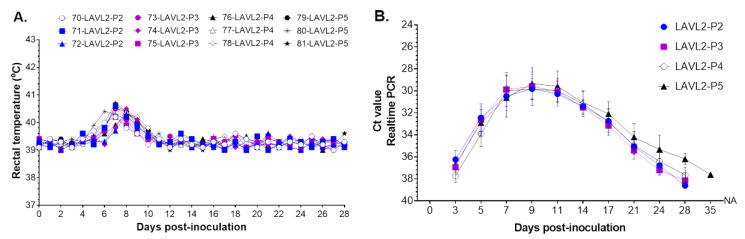
Safety of VNUA-ASFV-LAVL2 strain when passaged in pigs. (**A**) shows rectal temperatures of pigs from P2 to P5. (**B**) shows Ct values of ASFV in blood samples of pigs from P2 to P5. Data are presented as mean ± SD for pigs in each group.

To further evaluate the safety of VNUA-ASFV-LAVL2, we performed its serial passaging in pigs determined with pooled serum samples, which showed the highest Ct with ASFV RT-PCR. In total, five passages (P1 to P5) in pigs were completed in this study. Pigs in all passages showed transient low fever (<40.6 °C) by 6–9 DPI, then showed normal body temperature during the observation period (Figure 4A), and no obvious ASFV-specific clinical signs were observed. All pigs maintained a good daily feed intake between 2 to 5 pounds and were healthy and showed 100% survival until the end of tests. Viremia study showed that replication of VNUA-ASFV-LAVL2 reached its peak Ct value of about 30 (about 10^4^ HAD_50_/mL) at 9 DPI, and then the Ct value rapidly decreased, and VNUA-ASFV-LAVL2 was absent in blood from 11 DPI to 35 DPI (Figure 4B). All passages showed similar viremia and clearance rate of VNUA-ASFV-LAVL2 in inoculated pigs. 

### 3.5. Pigs Vaccinated with VNUA-ASFV-LAVL2 Were Fully Protected from Contemporary Pandemic ASFV Challenge

**Figure 5:**
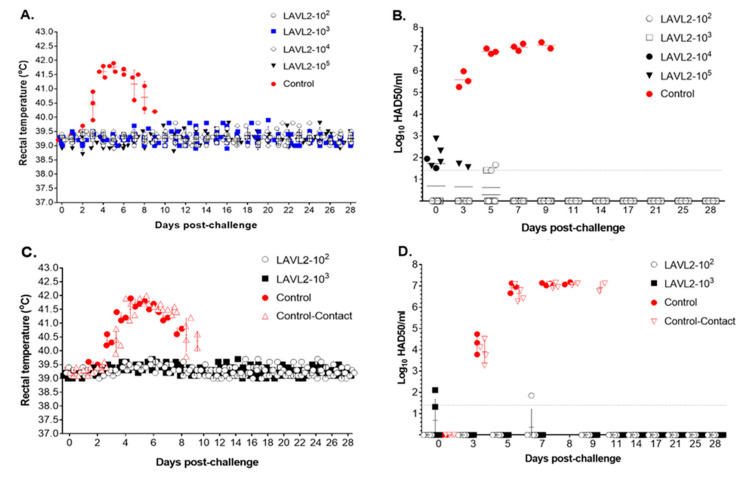
Rectal temperature and viremia of pigs after being challenged with virulent VNUA-ASFV-05L1 genotype II strain. (**A**) Rectal temperature of vaccinated and non-vaccinated control pigs after being challenged with standard dose of virulent VNUA-ASFV-05L1 strain. (**B**) Viremia of vaccinated and non-vaccinated control pigs after being challenged with standard dose of virulent VNUA-ASFV-05L1 strain. (**C**) Rectal temperature of vaccinated, non-vaccinated, and contact pigs after being challenged with a high dose of virulent VNUA-ASFV-05L1 strain. (**D**) Viremia of vaccinated, non-vaccinated, and contact pigs after being challenged with a high dose of virulent VNUA-ASFV-05L1 strain. The dotted line represents the limit of detection.

To test the efficacy of VNUA-ASFV-LAVL2, pigs in both vaccinated groups and the control group (inoculated with DMEM) were challenged with parental VNUA-ASFV-05L1, the contemporary pandemic genotype II ASFV. After challenge, pigs in the control group rapidly displayed clinical signs of ASF (from 5 to 9 DPC), including high fever (Figure 5A), anorexia, cough, depression, staggering gait, and diarrhea, and died from 8 to 9 DPC. In contrast, pigs vaccinated with VNUA-ASFV-LAVL2 (from low dose of 10^2^ HAD_50_ to high dose of 10^5^ HAD_50_) did not show elevated body temperatures after challenge (Figure 5A). The vaccinated pigs exhibited a high level of protection with 100% survival and were found to be healthy (without clinical signs) when challenged with virulent VNUA-ASFV-05L1. Remarkably, only a very low level of ASFV was detected in the blood before 5 DPC (Figure 5B), and ASFV was not detected in oral fluid or rectal swabs of all vaccinated groups. However, in the control group, the ASFV was detected in blood samples from 3 to 5 DPC, and it rapidly increased from 5 to 9 DPC (Figure 5B). 

To further test the protective efficacy of vaccination, we used a higher challenge dose (8 × 10^3^ HAD_50_) than the standard challenge dose for the VNUA-ASFV-LAVL2-vaccinated pigs (10^2^ and 10^3^ HAD_50_/dose). In addition, we housed two contact control pigs of each pig group in the same cage. The results showed that all vaccinated groups induced full protection, with 100% survival, and all pigs in those groups were found to be healthy when challenged with high doses of virulent VNUA-ASFV-05L1 strain (Figure 5C). Almost no ASFV was detected in blood (Figure 5D), oral fluid, and rectal swab samples of vaccinated pigs during the 28-day period of challenge. However, ASFV was detected in blood samples of control pigs and contact control pigs at 3 DPC, and it reached its highest titer from 7 to 9 DPC. The pigs from control group and contact control group died between 7 to 9 DPC. 

### 3.6. VNUA-ASFV-LAVL2 Vaccination Prevents ASFV Replication and Pathological Lesions in Pigs

High titers (Ct value from 14.15 to 27.81) of ASFV were detected in multiple organs (brain, heart, lung, liver, stomach, spleen, kidney, bladder, tonsil, lymph node, and bone marrow) of the control pigs at 7–9 DPC (Figure 5 and Table 2). In contrast, pigs vaccinated with VNUA-ASFV-LAVL2 (with doses from 10^2^ HAD_50_ to 10^5^ HAD_50_) had undetectable ASFV DNA in their organs, which was determined using real-time PCR analysis (Table 2) at 28 DPC. 

**Figure 6:**
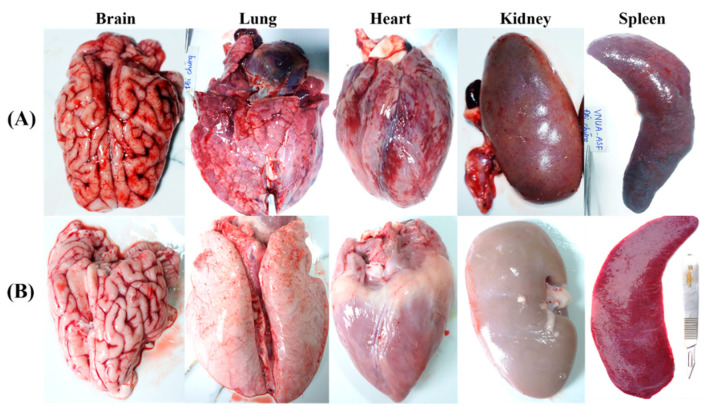
Pathological lesions found post-challenge in the organs of (**A**) vaccinated and (**B**) non-vaccinated pigs during necropsy.

Post-mortem examination showed that non-immunized control pigs displayed characteristic lesions and pathological signs of acute ASFV infection [30]. Severe macroscopic lesions (necrosis and hemorrhage) were observed in multiple organs including mandibular, pulmonary system, mesentery in the splenic lymph nodes, renal cortex, pericardium, myocardium, and cerebral meninges, with dark and enlarged spleen, along with swollen liver and gall bladder and interstitial pulmonary oedema in non-immunized control pigs (Figure 6A). In contrast, VNUA-ASFV-LAVL2-immunized pigs exhibited no clinical and pathological signs or lesions in any of these organs (Figure 6B).

### 3.7. Pigs Vaccinated with VNUA-ASFV-LAVL2 Developed ASFV-Specific Antibodies after Vaccination and Challenge

**Figure 7:**
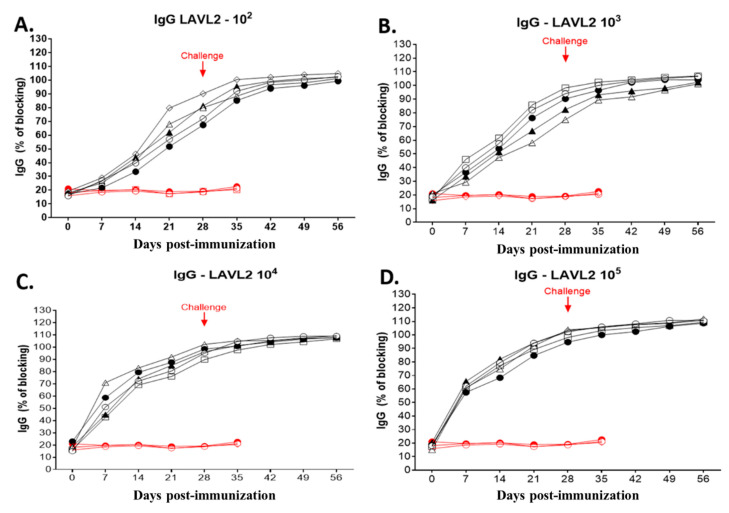
ASFV-specific antibodies were detected with ELISA only in pigs immunized with VNUA-ASFV-LAVL2. (**A**) ASFV-specific antibodies in serum samples of pigs immunized with 10^2^ HAD_50_ of VNUA-ASFV-LAVL2 and control pigs. (**B**) ASFV-specific antibodies in serum samples of pigs immunized with 10^3^ HAD_50_ of VNUA-ASFV-LAVL2 and control pigs. (**C**) ASFV-specific antibodies in serum samples of pigs immunized with 10^4^ HAD_50_ of VNUA-ASFV-LAVL2 and control pigs. (**D**) ASFV-specific antibodies in serum samples of pigs immunized with 10^5^ HAD_50_ of VNUA-ASFV-LAVL2 and control pigs. Black lines represent the pigs vaccinated with VNUA-ASFV-LAVL2. Red lines represent the non-vaccinated control pigs.

As shown in Figure 7, all vaccinated pigs developed ASFV-specific antibody after immunization with VNUA-ASFV-LAVL2. ASFV-specific antibodies were detected at 21 DPI (*p* < 0.05) in pigs immunized with a low dose of VNUA-ASFV-LAVL2 (10^2^ HAD_50_ and 10^3^ HAD_50_) (Figure 7A,B), and at 7–14 DPI (*p* < 0.01) in those immunized with a high dose of VNUA-ASFV-LAVL2 (10^4^ HAD_50_ and 10^5^ HAD_50_) (Figure 7C,D). At 28 DPI, the blocking percentage (%) of ASFV-specific antibodies reached approximately 87–102%, whereas no ASFV specific antibodies were detected in the control animals prior to the challenge with VNUA-ASFV-05L1. After the challenge with VNUA-ASFV-05L1, pigs tested positive for ASFV-specific antibodies till the end of challenge’s observation period. ASFV-specific antibodies were not detected in control pigs after the challenge with VNUA-ASFV-05L1. 

### 3.8. VNUA-ASFV-LAVL2 Induced Cellular Immunity in Vaccinated Pigs

**Figure 8:**
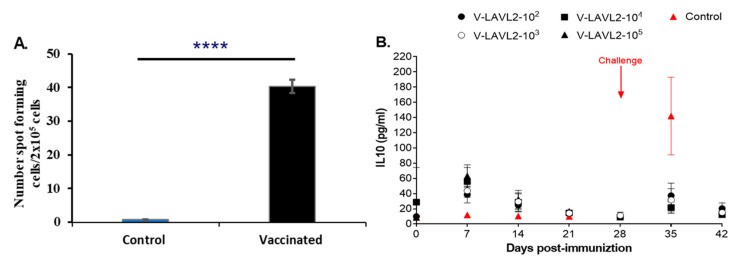
Cellular responses of VNUA-ASFV-LAVL2-vaccinated pigs and control pigs. (**A**) ELISPOT testing of ASFV-specific IFN-γ-producing PBMCs at 28 DPI. (**B**) ELISA testing to determine the serum IL-10 level of VNUA-ASFV-LAVL2-vaccinated pigs and control pigs during the vaccination period (Day 0 to Day 28) and challenge period (Day 28 to Day 42). *p*-values were determined using one-way ANOVA (**** *p* < 0.0001).

ELISPOT assay results showed that PBMCs of VNUA-ASFV-LAVL2-vaccinated pigs produced IFN-γ after stimulation with the wild-type VNUA-ASFV-05L1. The numbers of spot-forming cells from VNUA-ASFV-LAVL2-vaccinated pigs were significantly (*p* < 0.0001) higher than the cells from non-vaccinated pigs (Figure 8A). Cytokine IL-10 ELISA analysis showed that vaccination with VNUA-ASFV-LAVL2 induced the expression of IL-10 in pig serum, and it reached its peak at 7 DPI. However, after challenge with the wild-type VNUA-ASFV-05L1, non-vaccinated control pigs showed significantly higher serum IL-10 levels than those of pigs vaccinated with VNUA-ASFV-LAVL2, and these levels reached their peak at 7 DPC (35 DPI) (Figure 8B). 

## 4. Discussion

With the aim of developing a safe and efficacious ASF vaccine, we attempted to generate LAVs using cell passage. 3D4/21 cell line is derived from a single cell clone of 3D4 parent (a continuous porcine alveolar macrophages cell line). It has been reported that 3D4/21 can support the growth of cell-adapted ASFV-Lisbon 61 and field isolate Lillie SI/85 [31,32], but it was unable to maintain replication of the genotype II ASFV-HLJ/18 strain [33]. However, a more recent study showed that the ASFV genotype II strain CN/GS/2018 can attach and enter 3D4/21 cells, resulting in genome replication rate of up to 10^6^ copies/mL [34]. Our result is consistent with this study. The cell-adapted genotype II ASFVs can infect and replicate efficiently in 3D4/21 with a final concentration of 1.25% DMSO in the culture medium. We have developed several different LAV candidates using this technology and tested their safety in experimental pigs. 

VNUA-ASFV-LAVL2, derived from the field isolate (VNUA-ASFV-05L1, genotype II), showed one of the best attenuated phenotypes, and the best ability to induce protective immunity in pigs. Whole genome sequencing and analysis at passage 120 showed that VNUA-ASFV-LAVL2 harbored d 13 known gene deletions and 14 uncharacterized sequence deletions in the MGF region of VNUA-ASFV-LAVL2 (Table 1). VNUA-ASFV-LAVL2 can efficiently grow in both PAMs and 3D4/21 cells, and it showed comparable growth kinetics with less than 0.5 log_10_ at each time point post-infection compared to that of parental VNUA-ASFV-05L1 strain (Figure 1). This phenomenon indicates that the deleted known genes and uncharacterized sequences play a less crucial role in the ASFV replication. Mutations or deletions in the MGF region have been linked to the replication ability and the virulence attenuation of ASFV in both cell culture and pigs [19,20,21,35,36]. Therefore, it is not surprising that the deletion of these known MGF genes may play an important role in the attenuated phenotype of VNUA-ASFV-LAVL2 in experimental pigs. However, the replication and attenuation of VNUA-ASFV-LAVL2 in 3D4/21 cells may be associated with other deletions or mutations in the whole genome under selective pressures during the passaging of cells. To clarify the molecular mechanisms involved in the replication and attenuation of VNUA-ASFV-LAVL2, further investigations on the comparison of the whole genome sequences of different passages of VNUA-ASFV-LAVL2 in 3D4/21 cells and their virulence in pigs are needed. 

A variety of strategies have been described for developing ASFV LAVs that are able to persist for an extended period in host blood and tissues following immunization, and a correlation in terms of protection was found between enhanced clearance of virulent ASFVs and persistence of LAVs [12,13,23]. However, a potential drawback of using these LAVs in animals is the possibility of incomplete clearance of the vaccine strains that could result in reversion-to-virulence. Our findings from this study were particularly encouraging because although both the low doses (10^2^ and 10^3^ HAD_50_) and the high doses (10^4^ and 10^5^ HAD_50_) of VNUA-ASFV-LAVL2 were significantly attenuated and rapidly cleared from the blood, oral fluids, and rectal feces of pigs, the pigs were still able to sustain a protective immunity as evident from the challenge studies. VNUA-ASFV-LAVL2 was eliminated from blood by 28 DPI (Figure 2), and from feces or oral fluids by 17 DPI (Figure 3). Post-mortem examination showed that organs of VNUA-ASFV-LAVL2-immunized pigs showed no clinical and pathological signs or lesions (Figure 6). RT-PCR testing showed that VNUA-ASFV-LAVL2 was undetectable in these organs (Table 2). These results collectively make VNUA-ASFV-LAVL2 an attractive and promising LAV candidate from both safety and efficacy perspectives. We further tested the safety of VNUA-ASFV-LAVL2 by passaging it in pigs. The serum samples with the highest ASFV RT-PCR Ct value observed for vaccinated pigs were pooled and inoculated into pigs for passaging. VNUA-ASFV-LAVL2 remained safe and exhibited an attenuated phenotype after five passages in pigs. The main reason for using serum sample instead of whole blood in the serial in vivo passage experiment in this study was our concern about the potential blood-related side effects (such as allergic reaction, fever, and so on), which may affect the observation of ASFV-related clinical signs. However, serum samples only contain parts of ASFVs produced in the pigs. In future research, we will use the pool of whole blood samples and suspension of tissues to further test the safety (reversion-to-virulence) of VNUA-ASFV-LAVL2 in accordance with the guideline 41 of International Cooperation on Harmonisation of Technical Requirements for Registration of Veterinary Medicinal Products for the examination of live veterinary vaccines in target animals for the absence of reversion-to-virulence (reference number: EMA/CVMP/VICH/1052/2004). 

To identify factors of protective immunity that could be used to predict the vaccine efficacy of VNUA-ASFV-LAVL2, we tested the humoral and cellular immune responses of pigs immunized with VNUA-ASFV-LAVL2. Pigs immunized with VNUA-ASFV-LAVL2 are capable of inducing ASFV-specific IgG antibodies. Studies have speculated that cellular immune responses may play an important role in ASFV protective immunity. In particular, a T helper cell type 1 (Th1) immune response (producing IFN-γ) seems crucial in the establishment of a protective response against ASFV [37,38,39]. In this study, pigs vaccinated with VNUA-ASFV-LAVL2 possessed a significantly higher number of ASFV-specific IFN-γ-producing cells than non-vaccinated pigs. This observation is consistent with the previous reports [37,38,39]. 

## Figures and Tables

**Table 1:** Mutations of VNUA-ASFV-LAVL2 using the parental virus VNUA-ASFV-05L1 as a reference sequence.

Genome Position (bp)	Mutation Type	Gene	Nucleotide Change	Amino Acid Change	Protein Change
3797	Deletion	MGF360-18R	G	P to R	Disruption
6804	Substitution	I7L	T to A	Y to N	Single amino acid substitution
12,450	Substitution	I196L	T to C	I to I	No change
17,832	Deletion	I267L	A	K to R	Longer protein
21,502 to 21,503	Substitution	E199L	AG to GA	R to E	Single amino acid substitution
25,690	Substitution	E184L	A to G	N to D	Single amino acid substitution
40,293	Substitution	S237R	A to G	M to T	Single amino acid substitution
51,970	Substitution	NP868R	T to C	Q to R	Single amino acid substitution
73,715	Substitution	G1211R	A to G	V to A	Single amino acid substitution
84,062	Deletion	Uncharacterized	A	Uncharacterized	Uncharacterized
99,910	Substitution	C315R	A to G	V to A	Single amino acid substitution
105,303	Substitution	C717R	A to G	V to A	Single amino acid substitution
108,102	Substitution	Uncharacterized	T to C	Uncharacterized	Uncharacterized
113,352	Substitution	EP402R	T to C	Y to C	Single amino acid substitution
123,504	Substitution	K78R	T to C	N to S	Single amino acid substitution
133,757	Substitution	A179L	T to C	M to T	Single amino acid substitution
140,235	Deletion	A104R	T	K to S	Disruption
147,561	Substitution	MGF505-6R	A to G	V to A	Single amino acid substitution
161,147	Deletion	Uncharacterized	A	Uncharacterized	Uncharacterized
161,392	Deletion	MGF360-10L	A	E to D	Disruption
166,165	Deletion	MGF300-2R	G	D to I	Disruption
168,120 to168,159	Deletion	Uncharacterized	Deletion	Deletion	Deletion
168,160 to168,369	Deletion	X69R	Deletion	Deletion	Deletion
168,370 to 169,247	Deletion	Uncharacterized	Deletion	Deletion	Deletion
169,248 to 170,375	Deletion	MGF360-6L	Deletion	Deletion	Deletion
170,376 to 171,191	Deletion	Uncharacterized	Deletion	Deletion	Deletion
171,192 to 172,355	Deletion	MGF360-4L	Deletion	Deletion	Deletion
172,356 to 172,535	Deletion	Uncharacterized	Deletion	Deletion	Deletion
172,536 to 173,360	Deletion	MGF110-13L	Deletion	Deletion	Deletion
173,361 to173,445	Deletion	Uncharacterized	Deletion	Deletion	Deletion
173,446 to 173,805	Deletion	MGF110-12L	Deletion	Deletion	Deletion
173,806 to 173,994	Deletion	Uncharacterized	Deletion	Deletion	Deletion
173,995 to 174,360	Deletion	MGF110-14L	Deletion	Deletion	Deletion
174,361 to 174,449	Deletion	Uncharacterized	Deletion	Deletion	Deletion
174,450 to 174,809	Deletion	MGF110-11L	Deletion	Deletion	Deletion
174,810 to 175,099	Deletion	Uncharacterized	Deletion	Deletion	Deletion
175,100 to 175,972	Deletion	MGF110-9L	Deletion	Deletion	Deletion
175,973 to 176,130	Deletion	Uncharacterized	Deletion	Deletion	Deletion
176,131 to 176,505	Deletion	MGF100-1R	Deletion	Deletion	Deletion
176,506 to 176,722	Deletion	Uncharacterized	Deletion	Deletion	Deletion
176,723 to 177,106	Deletion	MGF110-8L	Deletion	Deletion	Deletion
177,107 to 177,234	Deletion	Uncharacterized	Deletion	Deletion	Deletion
177,235 to 177,519	Deletion	285L	Deletion	Deletion	Deletion
177,520 to 177,833	Deletion	Uncharacterized	Deletion	Deletion	Deletion
177,834 to 178,247	Deletion	MGF110-7L	Deletion	Deletion	Deletion
178,248 to 178,453	Deletion	Uncharacterized	Deletion	Deletion	Deletion
178,454 to 179,071	Deletion	MGF110-5L-6L	Deletion	Deletion	Deletion
179,072 to 179,259	Deletion	Uncharacterized	Deletion	Deletion	Deletion
179,260 to 179,634	Deletion	MGF110-4L	Deletion	Deletion	Shorter protein

**Table 2:** Detection of ASFV in organs of vaccinated and control pigs after challenge.

Groups	Pig No.	Challenge Dose	Real-Time PCR (Ct Value)												
			Brain	Heart	Lung	Liver	Stomach	Spleen	Kidney	Bladder	Tonsil	ILN	MLN	SLN	BM
**10^2^ HAD_50_ vaccinated**	28	1 × 10^3^ HAD_50_ of VNUA-ASFV-05L1	-	-	-	-	-	-	-	-	-	-	-	-	-
	29		-	-	-	-	-	-	-	-	-	-	-	-	-
	30		-	-	-	-	-	-	-	-	-	-	-	-	-
**10^3^ HAD_50_ vaccinated**	41		-	-	-	-	-	-	-	-	-	-	-	-	-
	42		-	-	-	-	-	-	-	-	-	-	-	-	-
	43		-	-	-	-	-	-	-	-	-	-	-	-	-
**10^4^ HAD_50_ vaccinated**	46		-	-	-	-	-	-	-	-	-	-	-	-	-
	48		-	-	-	-	-	-	-	-	-	-	-	-	-
	49		-	-	-	-	-	-	-	-	-	-	-	-	-
**10^5^ HAD_50_ vaccinated**	53		-	-	-	-	-	-	-	-	-	-	-	-	-
	54		-	-	-	-	-	-	-	-	-	-	-	-	-
	55		-	-	-	-	-	-	-	-	-	-	-	-	-
**Controls**	36		22.56	18.35	17.29	19.62	25.31	14.87	18.39	26.53	21.69	19.36	22.04	18.65	26.75
	37		21.75	20.47	18.23	21.29	24. 37	15.31	20.62	24.87	23.59	21.29	22.59	19.54	27.81
	38		21.18	19.59	18.62	20.47	25.59	14.15	19.57	25.18	21.37	20.57	23.18	20.47	27.17

Note: ILN: inguinal lymph node; MLN: mesenteric lymph node; SLN: submaxillary lymph node; BM: bone marrow; -: negative result for ASFV DNA with real-time PCR.

## Data Availability

All relevant data to support the findings described in the text are included in the main text. Additional data are available from the corresponding author upon reasonable request.
